# Effects of feeding high-moisture corn on performance, meat quality, and cecal microbiota of Jinling Yellow chickens

**DOI:** 10.3389/fvets.2026.1891207

**Published:** 2026-08-17

**Authors:** Tong Li, Buweiaizhar Maimaitimin, Linhai Song, Subinur Abduli, Yue Liu, Yiran Wang, Nuerminamu Aihemaiti, Wei Shao, Liang Yang, Wanping Ren

**Affiliations:** College of Animal Science, Xinjiang Agricultural University, Urumqi, China

**Keywords:** cecal microbiota, high-moisture corn, Jinling Yellow chickens, meat quality, muscle metabolomics, performance

## Abstract

This study aimed to investigate the effects of high- moisture corn (HMC) on the performance, meat quality, and cecal microbiota of Jinling Yellow chickens. A total of 216 healthy male Jinling Yellow chickens were randomly assigned to three dietary groups: a control group (CK, 100% dry cracked corn), an HMC50% group (50% dry cracked corn + 50% HMC), and an HMC100% group (100% HMC). Each group consisted of three replicate pens with 24 birds per pen, and the trial included a 7‑day adaptation period followed by a 60‑ day feeding period. On day 60, growth performance, slaughter traits, muscle nutrients, meat quality, untargeted metabolomics, and cecal 16S rRNA sequencing were evaluated. The HMC100% group showed significantly lower average daily gain (ADG) than the CK group (*p* < 0.05). The HMC50% group had significantly increased muscle valine, isoleucine, linoleic acid, and arachidonic acid (*p* < 0.05). Metabolomics identified 405 differential metabolites, with amino acid biosynthesis and glutathione metabolism significantly enriched, and high Ratio values for unsaturated fatty acid and glycerophospholipid pathways indicating lipid metabolism trends. The cecal *Firmicutes/Bacteroidetes* ratio increased, with *Weissella* enriched and *Lactobacillus* decreased; these microbial shifts correlated with fatty acid changes. These findings suggest that 50% HMC is the optimal inclusion level, which reshapes the cecal microbiota and improves meat quality through modulation of host lipid and amino acid metabolism.

## 1 Introduction

In broiler production, energy feed is a key factor determining production costs, with corn being an indispensable core energy feed, accounting for approximately 60–70% of the total feed cost (1). Nevertheless, the broiler industry continues to face challenges posed by high corn prices and limited feed resources; the conventional use of dry corn is associated with issues such as dust loss during grinding and processing, as well as mold contamination during storage, which not only leads to nutrient loss and economic inefficiency but also poses a serious threat to poultry health due to the production of mycotoxins, including aflatoxins and zearalenone, these contaminants can impair growth performance and immune function in poultry (2, 3). Therefore, selecting an efficient, low-cost, and safe approach to utilizing corn resources is of great significance for the sustainable development of the broiler industry. High-moisture corn (HMC) is well-established in ruminant production, and its positive effects on improving production performance and feed efficiency have been widely demonstrated. (4, 5) However, relevant studies on monogastric animals such as broilers remain scarce, and its underlying regulatory mechanisms have not been fully elucidated. Kunishige (6) and He (7) demonstrated that HMC can modulate intestinal microbiota and serum biochemical parameters by altering its physical morphology and biochemical properties, thereby improving growth performance and meat quality in poultry. Cruz-Polycarpo (8) reported that partial dietary replacement with HMC maintained or improved broiler growth performance, while Kunishige (6) confirmed its safety when applied in laying hens.

Jinling Yellow broiler is a Chinese meat-type chicken breed and a national-level livestock and poultry synthetic line in China. Developed using recessive white-feathered broilers, dwarf yellow chickens and Guangxi Sanhuang chickens as breeding materials, this strain was established through four generations of specialized line selection and cross combining ability tests, with a market age ranging from 80 to 120 days. As an important elite broiler breed in China, Jinling Yellow broiler is closely associated with Xinjiang signature dish, Xinjiang Big Plate Chicken. Favored by consumers for its delicious meat quality, approximately 50 million Jinling Yellow broilers are marketed in Xinjiang annually, generating an annual output value of around 190 million yuan and exhibiting great popularization and application prospects. Therefore, Jinling Yellow broilers were selected as the experimental animal in this study. In conclusion, this study investigated the effects of dietary replacement with HMC on growth performance, breast meat quality, and cecal microbiota of Jinling Yellow broilers, aiming to provide a scientific nutritional regulation strategy for industrialized, large-scale, and standardized broiler production.

## 2 Materials and methods

### 2.1 Animals

A total of 216 healthy Jinling Yellow chickens with similar age (50 ± 2 days) and uniform body weight (2.50 ± 0.10 kg) were selected as experimental subjects. All experimental procedures were performed in accordance with the *Guide for the Care and Use of Agricultural Animals in Research and Teaching (4th Edition, 2020)* (9) to avoid unnecessary discomfort of broilers. The animal care protocol for this experiment was approved by the Animal Ethics Committee of Xinjiang Agricultural University (Urumqi, China; Approval No. 20250620), and every effort was made to minimize animal suffering. At the end of the trial, all chickens were humanely euthanized via rapid cervical dislocation followed by exsanguination. No invasive surgical procedures were involved in this experiment.

### 2.2 Time and location

The experiment was conducted at a broiler farm in Hutubi County, Changji Hui Autonomous Prefecture, Xinjiang Uygur Autonomous Region. The entire project lasted from June 2025 to September 2025. Preliminary preparations, including animal selection, grouping, and feed formulation, were completed in July; subsequently, the core animal trial began, consisting of a 7-day acclimation period followed by a 60-day experimental feeding and data collection period. Samples collected on day 60 of the experimental phase were subjected to detection and analysis, with subsequent analytical work continuing until September.

### 2.3 Experimental design

A total of 216 healthy male Jinling Yellow chickens were randomly assigned to three dietary groups: a control group (CK, 100% dry cracked corn), an HMC50% group (50% dry cracked corn + 50% HMC), and an HMC100% group (100% HMC). In the HMC-supplemented groups, HMC was incorporated on an equal dry matter basis to replace the corresponding amount of dry cracked corn, each group consisted of three replicate pens, with 24 birds per pen. Each treatment group contained three replicate cages with 24 chickens per cage. Growth performance indicators including ADFI, ADG and FCR were statistically analyzed using cage replicates as experimental units (*n* = 3); individual birds served as experimental units for slaughter traits, meat quality, metabolomics, fatty acid, amino acid and cecal microbiota measurements. Cage replicate was incorporated as a random effect in the linear model for all statistical analyses. The experiment included a 7-day pre-feeding period, followed by a 60-day formal feeding period.

### 2.4 Feeding and management of experimental animals

During the experimental period, all broilers were housed in floor pens under identical management conditions, with natural lighting, an ambient temperature of 19–30 °C, and a relative humidity of 35–45%. Each pen measured 6.0 m × 4.0 m × 1.0 m and housed 24 birds, corresponding to a stocking density of 1.0 m^2^ per bird. Feed and water were provided ad libitum throughout the trial. The dietary formulations and nutrient levels of experimental chickens are presented in Table 1, while the physicochemical, nutritional and fermentation parameters (on a dry matter basis) of the HMC raw material are listed in Table 2:

**Table 1:** Dietary formula and nutrient levels (DM).

Ingredient	CK	HMC50%	HMC100%
Common corn (%)	60.00	30.00	0
HMC (%)	0	30.00	60.00
Soybean meal (%)	28.00	28.00	28.00
Wheat bran (%)	8.00	8.00	8.00
Additive premix ^1^ (%)	3.00	3.00	3.00
Calcium (%)	0.50	0.50	0.50
Salt (%)	0.50	0.50	0.50
Total	100	100	100
Nutrient levels			
Metabolizable energy (MJ/kg)	13.50	13.58	13.63
Crude protein (%)	17.02	17.14	17.20
Crude fat (%)	4.36	4.33	4.29
NDF (%)	17.13	25.86	28.73
ADF (%)	11.99	18.10	20.11
Phosphorus (%)	0.37	0.36	0.36

^1^The premix provided per kilogram of feed: VA 12,000 IU, VD₃ 3,000 IU, VE 20 IU, VK₃ 3 mg, VB₁ 3 mg, VB₂ 8 mg, VB₆ 6 mg, VB₁₂ 0.05 mg, D-pantothenic acid 20 mg, niacin 50 mg, biotin 0.2 mg, folic acid 1.5 mg, choline 1,000 mg, Cu 10 mg, Zn 110 mg, Fe 100 mg, Mn 100 mg, Se 0.16 mg, I 0.6 mg.

**Table 2:** Nutrient composition of major ingredients in the HMC (DM).

Ingredient	HMC
DM(%)	74.10
Crude protein (%)	8.66
Crude fat (%)	4.31
Starch (%)	65.40
Lysine (%)	0.28
Methionine (%)	0.17
Calcium (%)	0.03
Phosphorus (%)	0.09
pH	4.20
Lactic acid (% of DM)	4.30
Acetic acid (% of DM)	0.40

### 2.5 Sample collection and indicator measurement

In this experiment, three dietary treatment groups were included to evaluate the gradient effects on growth performance; but only the CK and HMC50% groups were used for slaughter sampling and further analysis, including fatty acid and amino acid profiling, muscle metabolome, and cecal microbial community sequencing. This study aimed to identify the optimal replacement proportion of high-moisture corn that simultaneously improves meat quality and delivers economic benefits for broiler production. Broilers fed the 100% HMC diet exhibited markedly impaired growth performance throughout the feeding period, rendering this treatment unsuitable for large-scale commercial application; it was only retained as a negative gradient control group. To avoid scattering the core research focus of this paper, molecular mechanistic analyses were only performed on the optimal 50% HMC substitution group.

#### 2.5.1 Performance

On day 60 of the experimental period, 12 experimental chickens with body weight close to the average were selected from the CK group and the optimal experimental group (HMC50% group) for slaughter (4 chickens per replicate). Slaughter weight, eviscerated yield percentage, and dressing percentage were determined according to the *Terms and Measurement Statistics Methods for Poultry Production Performance* (NY/T823-2020) (10). The core objective of this trial was to elucidate the regulatory mechanisms of high-moisture corn on nutritional composition and molecular metabolic pathways in broiler breast muscle, accordingly, during the slaughter sampling stage, priority was given to collecting sufficient breast muscle tissue for subsequent analyses of fatty acids, amino acids, metabolomics, and cecal microbiota.

#### 2.5.2 Meat quality

Determination of muscle nutrient composition: crude protein (CP), crude fat (CF), pH, meat color (lightness L*, redness a*, yellowness b*), water-holding capacity, and shear force. The CP content was analyzed using a FOSS 2300 automatic nitrogen analyzer according to GB 5009.5–2010 *Determination of Protein in Foods* (11). The CF content was determined using a FOSS Soxtec system fat analyzer according to GB/T 14772–2008 *Determination of Crude Fat in Foods* (12). Amino acids were analyzed by Nanjing Jiancheng Bioengineering Institute (Nanjing, China), and fatty acids were analyzed by Beijing Novogene Technology Co., Ltd. (Beijing, China).

Meat quality measurements included: pH was measured using a pH meter (Field Scout Syoustik, Spectrum); water-holding capacity was measured using a meat water-holding capacity analyzer (MAEC-18, Ming’ao Instrument Equipment Co., Ltd.); shear force was determined using a digital meat tenderness meter (C-LM3B, College of Engineering, Northeast Agricultural University).

#### 2.5.3 Cecal microorganisms

On day 60 of the experiment, cecal content samples were collected from the slaughtered chickens into 2 mL sterile cryotubes and immediately frozen for storage. The cecal contents were sent to Beijing Novogene Technology Co., Ltd. (Beijing, China) for high-throughput sequencing of 16S rRNA fragments. The universal primer sequences used were 341F (5’-CCTAYGGGRBGCASCAG-3′) and 806R (5’-GGACTACNNGGGTATCTAAT-3′). The raw image data obtained from high-throughput sequencing were converted into raw sequencing reads through base calling analysis (13). Alpha diversity (Chao1 index, Shannon index, Simpson index) was calculated using QIIME software; species annotation of feature sequences was performed using QIIME2 combined with the SILVA database, and biological classification was carried out according to domain, phylum, class, order, family, and genus (14, 15).

### 2.6 Statistical analysis

Data were preliminarily processed using Excel 2016 (Microsoft Corp., Redmond, WA, USA), and statistical analysis was performed using SPSS 27.0 (software version 20.0, IBM, NY) with analysis of variance. The analytical methods included mixed linear models and independent samples *t*-tests. *p* < 0.05 was considered statistically significant, while *p* > 0.05 indicated no significant difference. All results are expressed as mean ± standard deviation (Mean ± SD).

Prior to correlation analysis, the Shapiro–Wilk normality test was performed on all fatty acid variables and relative abundances of cecal microbial genera, and all indicators conformed to a normal distribution (*p* > 0.05), therefore, parametric Pearson correlation analysis was used to evaluate the linear associations between cecal microbial genera and breast muscle fatty acids, whereas the non-parametric Spearman rank correlation analysis was applied when the data deviated from normal distribution.

## 3 Results and analysis

### 3.1 Effects of feeding HMC on the performance of Jinling Yellow chickens

The effects of Feeding HMC on the Performance of Jinling Yellow chickens were shown in Table 3, the HMC100% group exhibited a 16.91% higher feed conversion ratio (FCR) and a 14.82% lower average daily gain (ADG) than the CK group (*p* < 0.05); however, no significant differences were observed among the CK, HMC50%, and HMC100% groups in average daily feed intake, final body weight, eviscerated weight, or dressing percentage (*p* > 0.05).

**Table 3:** Effects of HMC on growth performance in Jinling Yellow chickens during the 60-day feeding period.

Item	CK	HMC50%	HMC100%	*P*-value
ADFI (g/d)	173.82	169.12	161.76	0.07
FCR	6.86^b^	7.20	8.02^a^	0.03
Initial weight (kg)	2.53 ± 0.25	2.56 ± 0.17	2.54 ± 0.26	0.21
Final weight (kg)	4.05 ± 0.40	3.97 ± 0.40	3.75 ± 0.39	0.20
ADG(kg)	0.027 ± 0.002^a^	0.026 ± 0.002^a^	0.023 ± 0.001^b^	0.04
Eviscerated weight (kg)	3.12 ± 0.23	3.10 ± 0.25	3.09 ± 0.26	0.80
Dressing percentage (%)	77.03 ± 1.79	78.09 ± 2.83	76.89 ± 2.53	0.31

^ab^Different lowercase superscript letters within the same row indicate significant differences (*P* < 0.05), and ^AB^different uppercase superscript letters within the same row indicate extremely significant differences (*P* < 0.01).

### 3.2 Effects of feeding HMC on meat quality of Jinling Yellow chickens

#### 3.2.1 Effects of feeding HMC on breast muscle quality indices of Jinling Yellow chickens

The effects of Feeding HMC on Breast Muscle Quality Indices of Jinling Yellow chickens were shown in Table 4, the fat content in the breast muscle of the HMC50% group was 15.94% higher than that of the CK group (*p* < 0.05), while shear force, water-holding capacity, and meat color lightness (*L*) were 42.30, 13.08, and 4.76% lower than those in the control group, respectively (*p* < 0.05). However, no significant differences were observed in protein content, redness (a*), or yellowness (b*) between the HMC50% group and the control group (*p* > 0.05).

**Table 4:** Effect of HMCorn on breast meat quality of Jinling Yellow chickens after a 60-day feeding experiment.

Item	CK	HMC50%	*P*-value
Fat content (%)	3.20 ± 0.02	3.71 ± 0.04	0.04
Protein content (%)	25.77 ± 1.66	27.04 ± 1.05	0.07
Shear force (N)	22.37 ± 2.13^a^	15.72 ± 2.13^b^	0.04
Water-holding capacity (%)	22.74 ± 1.41^a^	20.11 ± 1.53^b^	0.02
Meat color *L*	55.07 ± 2.53^a^	52.57 ± 2.39^b^	0.02
Meat color a*	2.35 ± 0.53	2.25 ± 0.53	0.84
Meat color b*	7.41 ± 0.87	7.37 ± 0.50	0.94

^ab^Different lowercase superscript letters within the same row indicate significant differences (*P* < 0.05), and ^AB^different uppercase superscript letters within the same row indicate extremely significant differences (*P* < 0.01).

#### 3.2.2 Effects of feeding HMC on fatty acids in the breast muscle of Jinling Yellow chickens

The effects of Feeding HMC on Fatty Acids in the Breast Muscle of Jinling Yellow chickens were shown in Table 5, the saturated fatty acid content in the muscle of the HMC50% group was 7.33% higher than that in the CK group (*p* > 0.05); the monounsaturated fatty acid content in the HMC50% group was 26.18% higher than that in the CK group (*p* < 0.05), among which the contents of *trans*-7-nonadecenoic acid and *cis*-11-eicosenoic acid were 96.85 and 547.78% higher than those in the CK group, respectively (*p* < 0.01); the polyunsaturated fatty acid content in the HMC50% group was 51.88% higher than that in the CK group (*p* < 0.05), among which the contents of linoleic acid, *cis*-11,14-eicosadienoic acid, *cis*-11,14,17-eicosatrienoic acid, and arachidonic acid were 51.64, 125.20, 33.14, and 58.28% higher than those in the CK group, respectively (*p* < 0.05); the total fatty acid content in the HMC50% group was 31.86% higher than that in the CK group (*p* < 0.01).

**Table 5:** Effect of HMC on breast muscle fatty acids in Jinling Yellow chickens after 60-day of feeding (ng/g).

Classification	Peak Name	CK	HMC50%	*P*-value
Saturated fatty acids (SFA)	Caprylic acid	170.35 ± 8.22	154.68 ± 8.56	0.22
	Decanoic acid	12.41 ± 0.51	12.93 ± 0.35	0.43
	Hendecanoic acid	33.83 ± 0.85	36.07 ± 1.68	0.26
	Dodecanoic acid	244.12 ± 16.81	254.60 ± 18.77	0.69
	Tridecanoic acid	47.30 ± 2.05	45.43 ± 2.08	0.54
	Tetradecanoic acid	636.35 ± 42.76	662.17 ± 38.34	0.66
	Pentadecanoic acid	395.26 ± 26.95	346.24 ± 20.83	0.18
	Hexadecanoic acid	24897.93 ± 2516.57	28433.32 ± 2553.09	0.35
	Heptadecanoic acid	328.71 ± 16.85	359.31 ± 30.12	0.40
	Octadecanoic acid	31484.26 ± 2439.55	32360.24 ± 1742.90	0.78
	Arachidic acid	409.12 ± 38.19	412.87 ± 26.09	0.94
	Heneicosanoic acid	22.21 ± 5.14	24.80 ± 0.67	0.63
	Docosanoic acid	140.89 ± 14.21	118.17 ± 24.36	0.44
	Tetracosanoic acid	244.19 ± 61.24	175.05 ± 12.37	0.30
Monounsaturated fatty acids (MUFA)	Myristoleic acid	21.39 ± 1.95	25.04 ± 3.54	0.39
	Myristelaidic acid	10.92 ± 2.03	14.45 ± 0.84	0.14
	*cis*-10-Pentadecenoic acid	10.56 ± 0.77	9.01 ± 1.53	0.39
	*trans*-10-Pentadecenoic acid	3.38 ± 1.61	3.34 ± 1.58	0.99
	Palmitoleic acid	670.77 ± 88.63	757.21 ± 102.69	0.54
	Palmitelaidic acid	100.64 ± 9.71	97.29 ± 12.76	0.84
	*cis*-10-Heptadecenoic acid	27.56 ± 1.77	30.68 ± 3.21	0.41
	*trans*-10-Heptadecenoic acid	40.73 ± 4.47	47.47 ± 5.17	0.35
	Oleic acid	11880.65 ± 1850.73	15101.78 ± 1542.55	0.21
	*cis*-Vaccenic acid	10614.65 ± 1642.33	13347.85 ± 1309.12	0.22
	*trans*-Vaccenic acid	229.21 ± 21.21	263.86 ± 17.87	0.24
	*trans*-7-Nonadecenoic acid	25.72 ± 1.94^b^	50.64 ± 6.26^a^	0.01
	*trans*-10-Nonadecenoic acid	7.97 ± 0.24	8.30 ± 0.99	0.75
	*cis*-11-Eicosenoic acid	22.13 ± 1.24^B^	143.36 ± 73.85^A^	<0.01
	*trans*-11-Eicosenoic acid	444.58 ± 60.58	577.79 ± 68.91	0.18
	Brassidic acid	183.13 ± 32.85	178.33 ± 20.91	0.90
	Nervonic acid	27.80 ± 4.24	32.40 ± 3.22	0.41
Polyunsaturated fatty acids (PUFA)	Linoleic acid	13286.92 ± 1426.07^b^	20149.73 ± 2294.84^a^	0.03
	Linoelaidic acid	82.68 ± 7.03	91.09 ± 6.34	0.40
	gamma-Linolenic acid	257.23 ± 20.81	320.25 ± 37.95	0.18
	alpha-Linolenic acid	354.82 ± 41.94	458.39 ± 58.23	0.18
	*cis*-11,14-Eicosadienoic acid	689.99 ± 152.16^b^	1553.73 ± 216.55^a^	0.01
	homo-gamma-Linolenic acid	2483.68 ± 274.82	3113.56 ± 515.39	0.31
	*cis*-11,14,17-Eicosatrienoic acid	3036.25 ± 176.81^b^	4042.17 ± 363.52^a^	0.03
	Arachidonic acid	43553.48 ± 6819.16^b^	68938.15 ± 6695.58^a^	0.02
	*cis*-13,16-Docosadienoic acid	95.03 ± 8.42	118.59 ± 12.83	0.16
	*cis*-5,8,11,14,17-Eicosapentaenoic acid	1277.33 ± 179.93	1526.84 ± 285.21	0.48
	*cis*-7,10,13,16-Docosic acidtraenoic acid*	7141.18 ± 853.16	9954.56 ± 1894.87	0.21
	*cis*-7,10,13,16,19-Docosapentaenoic acid	2278.43 ± 789.76	2940.04 ± 682.46	0.54
	*cis*-4,7,10,13,16-Docosapentaenoic acid	2867.69 ± 862.99	4689.36 ± 1228.91	0.25
	*cis*-4,7,10,13,16,19-Docosahexaenoic acid	1902.29 ± 1001.77	2552.25 ± 670.64	0.60
TFAC		162695.68 ± 19614.37^B^	214533.39 ± 19479.76^A^	<0.01

^ab^Different lowercase superscript letters within the same row indicate significant differences (*P* < 0.05), and ^AB^different uppercase superscript letters within the same row indicate extremely significant differences (*P* < 0.01).

#### 3.2.3 Effects of feeding HMC on amino acids in the breast muscle of Jinling Yellow chickens

The effects of Feeding HMC on Amino Acids in the Breast Muscle of Jinling Yellow chickens were shown in Table 6, the essential amino acid content in the muscle of the HMC50% group was 0.66% higher than that in the CK group (*p* > 0.05), among which the contents of Val and Ile were 15.09 and 14.51% higher, respectively (*p* < 0.05); the contents of non-essential and conditional amino acids in the HMC50% group showed no significant differences compared with the CK group (*p* > 0.05); the total amino acid content in the HMC50% group was 1.36% lower than that in the CK group (*p* > 0.05).

**Table 6:** Effect of HMC on breast muscle amino acids in Jinling Yellow chickens after 60-day of feeding (mg/g).

Classification	Peak Name	CK	HMC50%	*P*-value
Essential amino acid	Thr	10.68 ± 0.58	10.60 ± 0.76	0.83
	Val	9.01 ± 0.70^b^	10.37 ± 0.90^a^	0.02
	Met	5.49 ± 0.41	5.28 ± 0.62	0.49
	Ile	7.79 ± 0.46^B^	8.92 ± 0.70^A^	0.01
	Leu	18.29 ± 1.03	18.07 ± 1.94	0.81
	Phe	7.02 ± 0.59	7.66 ± 0.97	0.19
	His	17.01 ± 1.55	16.19 ± 1.97	0.42
	Lys	19.32 ± 1.36	18.81 ± 1.87	0.60
	Arg	13.88 ± 1.02	13.30 ± 1.46	0.45
Non-essential amino acid	Asp	23.57 ± 1.45	22.56 ± 1.48	0.26
	Ser	9.85 ± 0.48	9.39 ± 0.61	0.17
	Glu	38.87 ± 2.21	38.02 ± 2.61	0.56
	Gly	10.24 ± 0.41	9.86 ± 0.41	0.23
	Ala	14.18 ± 0.78	13.44 ± 1.13	0.22
	Pro	7.82 ± 0.39	7.07 ± 0.31	0.57
Conditional amino acids	Cys	3.65 ± 0.28	3.52 ± 0.43	0.55
	Tyr	6.04 ± 0.53	6.62 ± 0.81	0.17
TAA		222.71 ± 19.85	219.68 ± 21.38	0.37

^ab^Different lowercase superscript letters within the same row indicate significant differences (*P* < 0.05), and ^AB^different uppercase superscript letters within the same row indicate extremely significant differences (*P* < 0.01).

#### 3.2.4 Effects of feeding HMC on muscle metabolism in the breast muscle of Jinling Yellow chickens

##### 3.2.4.1 Principal component analysis (PCA)

**Figure 1:**
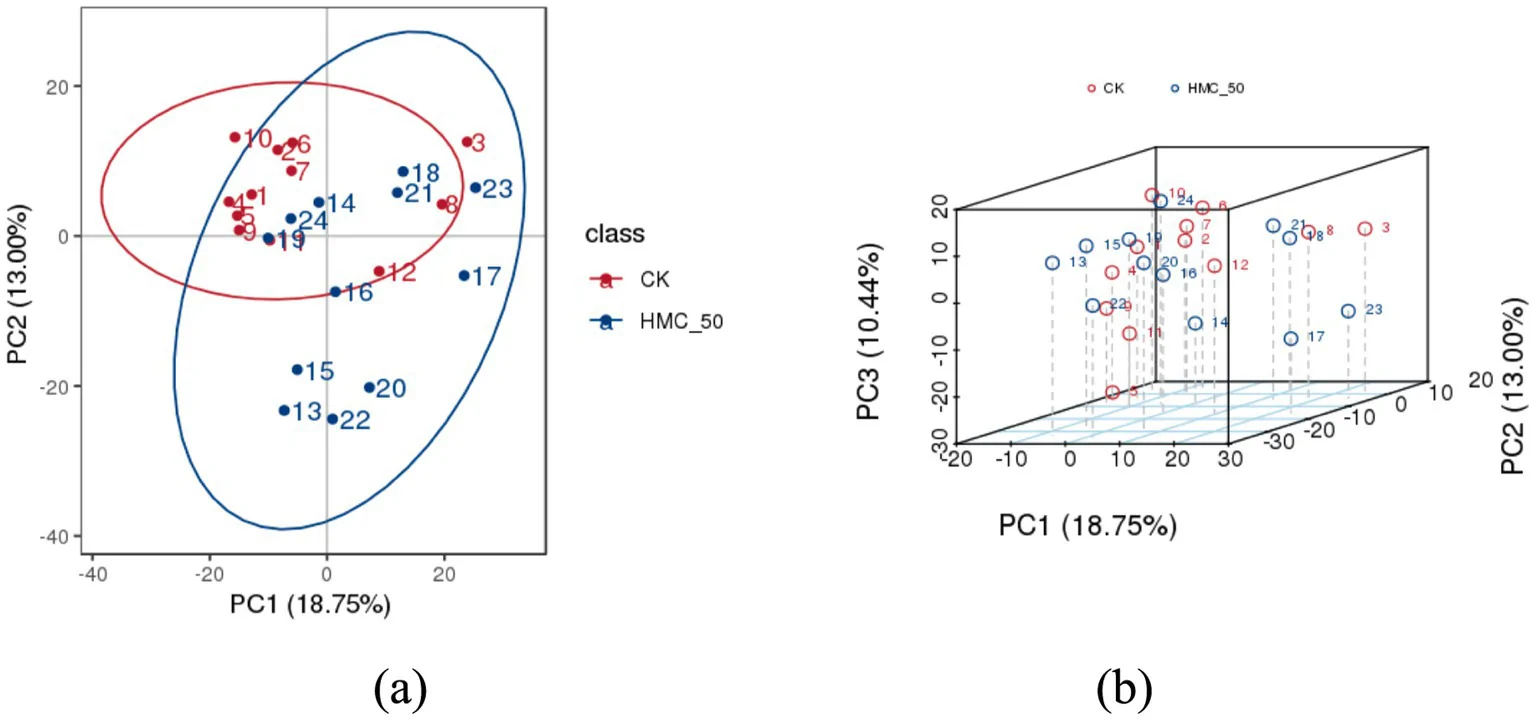
PCA analysis of muscle metabolism in Jinling Yellow chickens fed with HMC: Red (CK) represents the control group, and blue (HMC_50) represents the HMC 50% group. **(a)** Principal component analysis (PCA) score plot based on muscle metabolomics data in negative ion mode. Each point represents an individual sample. The *x*-axis (*PC1*) and *y*-axis (*PC2*) represent the scores of the first and second principal components, respectively. Scatter points in different colors indicate samples from different experimental groups: Red for the control group and blue for the experimental group. The ellipse indicates the 95% confidence interval. **(b)** Three-dimensional PCA score plot based on muscle metabolomics data, with samples projected onto the first three principal.

The result was shown in Figure 1a, in the negative ion mode, the first principal component (PC1) and the second principal component (PC2) explained for 18.75 and 13.00% of the total variance, respectively, the clear separation of the two groups along the PC1 axis indicated that the dietary intervention induced significant alterations in the overall metabolic profiles; as shown in 1-(b), the first, second, and third principal components (PC1, PC2, and PC3) explained 18.75, 13.00, and 10.44% of the total variance, respectively, illustrating the separation of the two groups in multidimensional space.

##### 3.2.4.2 Partial least squares discriminant analysis (PLS-DA)

**Figure 2:**
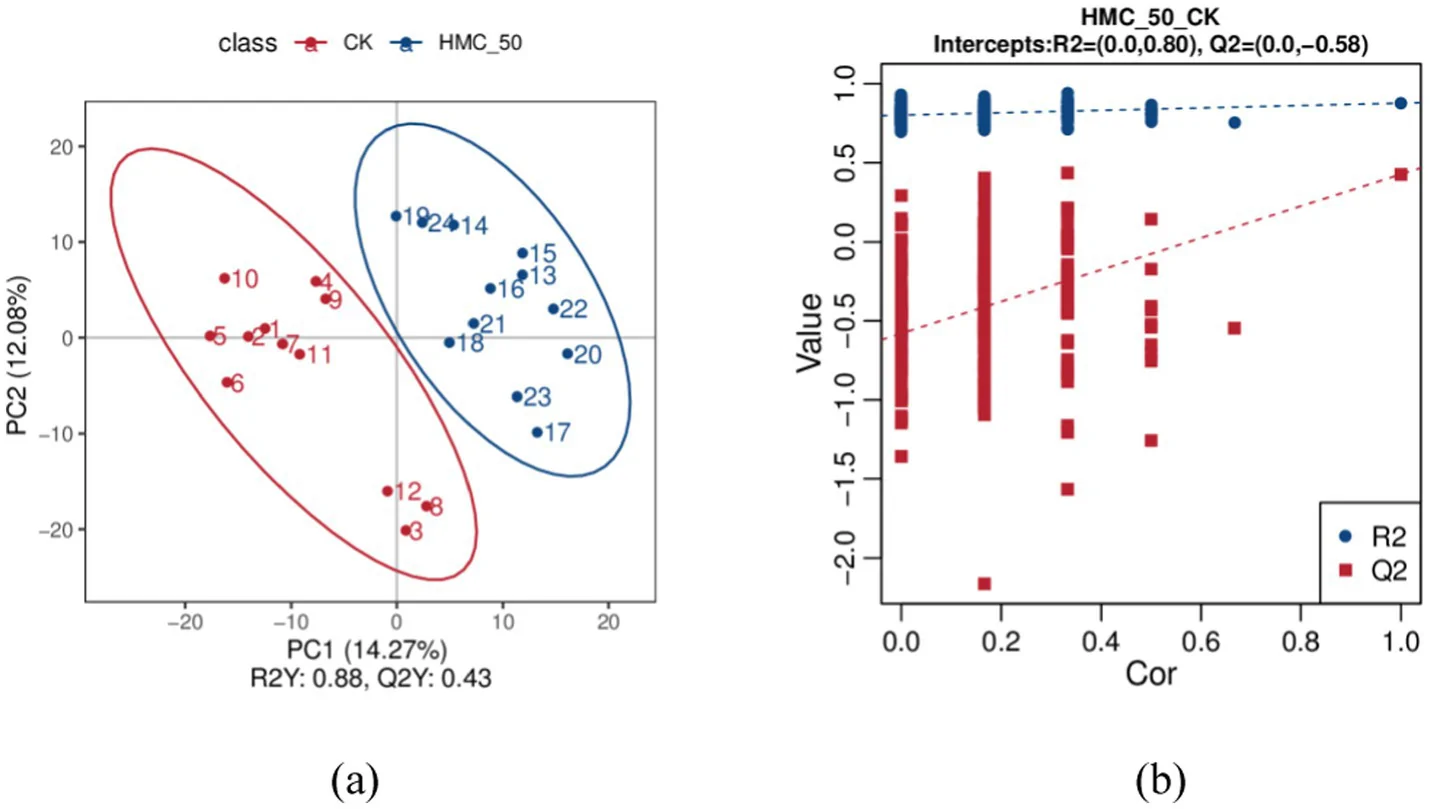
PLS-DA analysis of muscle metabolism in Jinling Yellow chickens fed with HMC: **(a)** Score scatter plot. Red (CK) represents the control group, and blue (HMC_50) represents the HMC 50% group. The *x*-axis indicates the scores of samples on the first principal component; The *y*-axis indicates the scores of samples on the second principal component; **(b)** Permutation test validation of the PLS-DA model based on 200 permutations, blue dots represent *R*^2^ values, and red squares denote *Q*^2^ values, the *Q*^2^-intercept from the permutation test was −0.58, indicating that the model was not overfitted and that the group discrimination was stable.

The result was shown in Figure 2a, in the negative ion mode, the first principal component (PC1) and the second principal component (PC2) explained 14.27 and 12.08% of the inter-group variance, respectively, indicating a clear separation of the two groups along the PC1 axis; the result was shown in Figure 2b, the PLS-DA model for the two groups yielded goodness-of-fit parameters *R^2^*Y = 0.88 and *Q^2^*Y = 0.43, and the *Q^2^*-intercept from the permutation test was −0.58 (< 0), indicating that the model was not overfitted and that the discrimination between the two groups was robust and reliable.

##### 3.2.4.3 Screening of differential metabolites

**Figure 3:**
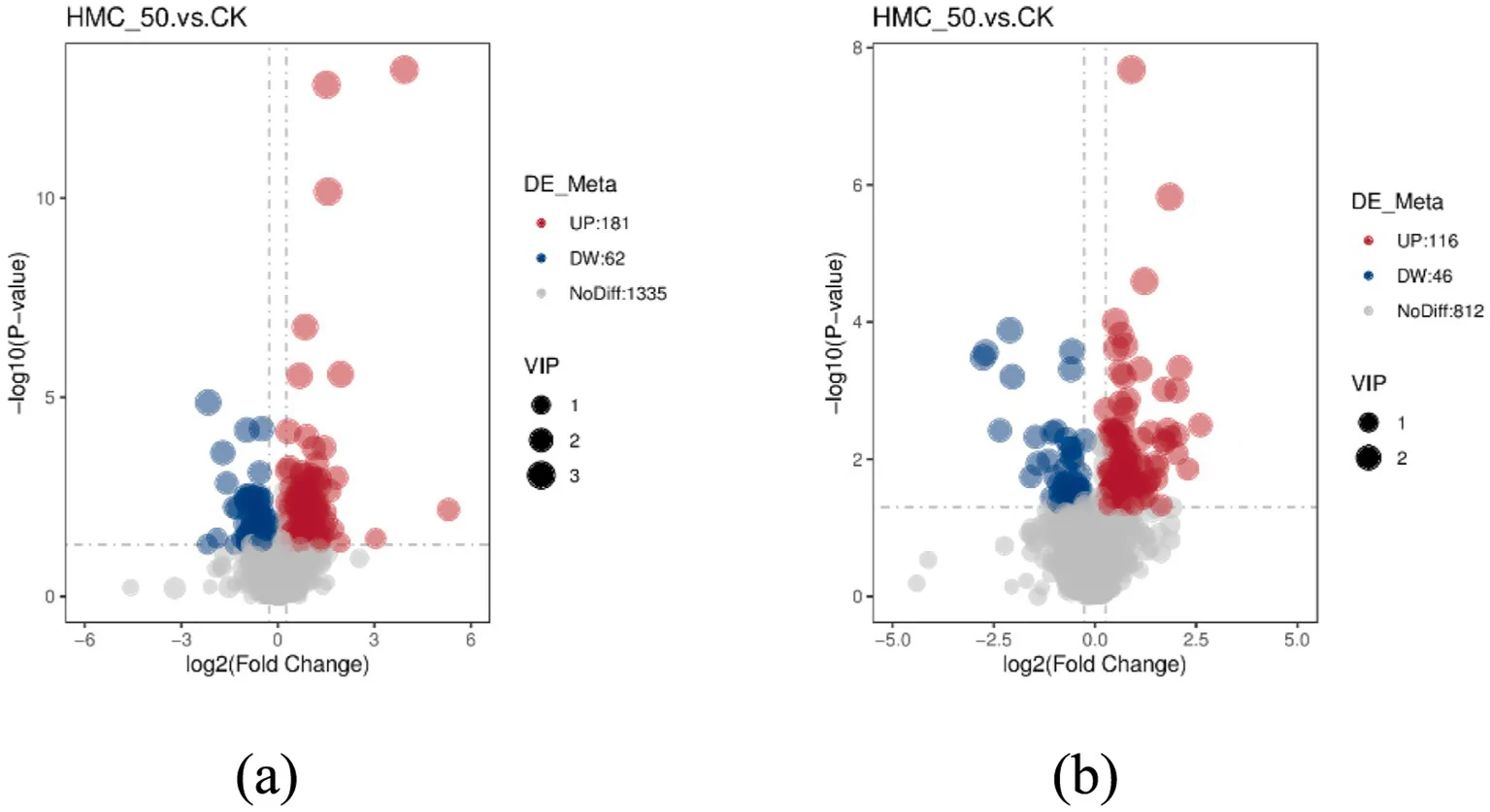
PLS-DA analysis of muscle metabolism in Jinling Yellow chickens fed with HMC: CK represents the control group, HMC_50 represents the HMC 50% group **(a)** Positive ion mode (POS), **(b)** Negative ion mode (NEG). Each point represents a metabolite. Gray points indicate no significant change; red points indicate significant upregulation (*VIP* > 1.0, *p* < 0.05, and FC > 1.2); blue points indicate significant downregulation (VIP > 1.0, *p* < 0.05, and *FC* < 0.833). The numbers of significantly upregulated and downregulated metabolites are indicated. The vertical dashed lines represent the fold change thresholds.

The results were shown in Table 7 and Figure 3, using thresholds of *p* < 0.05, VIP > 1.0, and FC > 1.2 or FC < 0.833, a total of 405 significant differential metabolites were screened. Among these, 243 differential metabolites were identified in positive ion mode (POS) (181 upregulated, 62 downregulated), and 162 differential metabolites were identified in negative ion mode (NEG) (116 upregulated, 46 downregulated). Table 8 presents the KEGG pathway enrichment analysis of differential metabolites screened in positive ion mode, the results revealed a total of six enriched metabolic pathways, among which glutathione metabolism, ferroptosis, arginine biosynthesis, and alanine/aspartate/glutamate metabolism were significantly enriched, whereas the biosynthesis of unsaturated fatty acids and glycerophospholipid metabolism, although not reaching statistical significance (*p* > 0.05), exhibited relatively high enrichment ratios (Ratio > 0.4). For the ferroptosis pathway (*p* = 0.005, Ratio = 1), four differential metabolites were involved, including glutathione, *L*-glutamate, and *L* (−)-glutathione. For glutathione metabolism (*p* = 0.024, Ratio = 0.6), in addition to the above four metabolites, cystinylglycine and 5-oxoproline were also included. The arginine biosynthesis pathway (*p* = 0.045, Ratio = 0.67) was enriched with *L*-glutamine, *L*-glutamate, and N-acetylornithine. The alanine, aspartate and glutamate metabolism pathway (*p* = 0.059, Ratio = 0.75) was enriched with *L*-asparagine, *L*-glutamine, and *L*-glutamate. The unsaturated fatty acid biosynthesis pathway (*p* = 0.121, Ratio = 0.60) contained four differential metabolites, namely eicosanoid, *cis*-5,8,11,14,17-eicosapentaenoic acid and *cis*-11,14,17-tricosatrienoic acid, et al. The glycerophospholipid metabolism pathway (*p* = 0.133, Ratio = 0.43) involved nine differential metabolites including cytidine diphosphate choline, choline and choline phosphate, et al.

**Table 7:** Differential metabolite screening results.

Ion mode	Total identified metabolites	Significantly upregulated number	Significantly downregulated number	Threshold
POS	243	181	62	*P* < 0.05, VIP > 1.0, FC > 1.2 or < 0.833
NEG	162	116	46	*P* < 0.05, VIP > 1.0, FC > 1.2 or <0.833

**Table 8:** KEGG enrichment pathway analysis.

Pathway	*P*-value	Ratio	Enriched metabolites
Ferroptosis	0.005	1.00	Glutathione; *L*-Glutamic acid; *L* (−)-Glutathione; g-Glutamylcysteine
Glutathione metabolism	0.024	0.60	Glutathione; *L*-Glutamic acid; *L* (−)-Glutathione; Cysteinylglycine; g-Glutamylcysteine; 5-oxoproline
Arginine biosynthesis	0.045	0.67	*L*-Glutamine; *L*-Glutamic acid; N-Acetylornithine; *L*-Arginine
Alanine, aspartate and glutamate metabolism	0.059	0.75	*L*-Asparagine; *L*-Glutamine; *L*-Glutamic acid
Biosynthesis of unsaturated fatty acids	0.121	0.60	*cis*-11-Eicosenoic acid; *cis*-5,8,11,14,17-Eicosapentaenoic acid; *cis*-11,14,17-Eicosatrienoic acid
Glycerophospholipid metabolism	0.133	0.43	sn-Glycero-3-phosphocholine; Citicoline; N-Methylethanolamine phosphate; Choline; LysoPC (P-18:0/0:0); LysoPC (16:1(9Z)/0:0); LysoPC (20:4(5Z,8Z,11Z,14Z)/0:0); LysoPC(22:4(7Z,10Z,13Z,16Z)/0:0); LysoPC(24:1(15Z)/0:0)

##### 3.2.4.4 Clustering heatmap of differential metabolites

**Figure 4:**
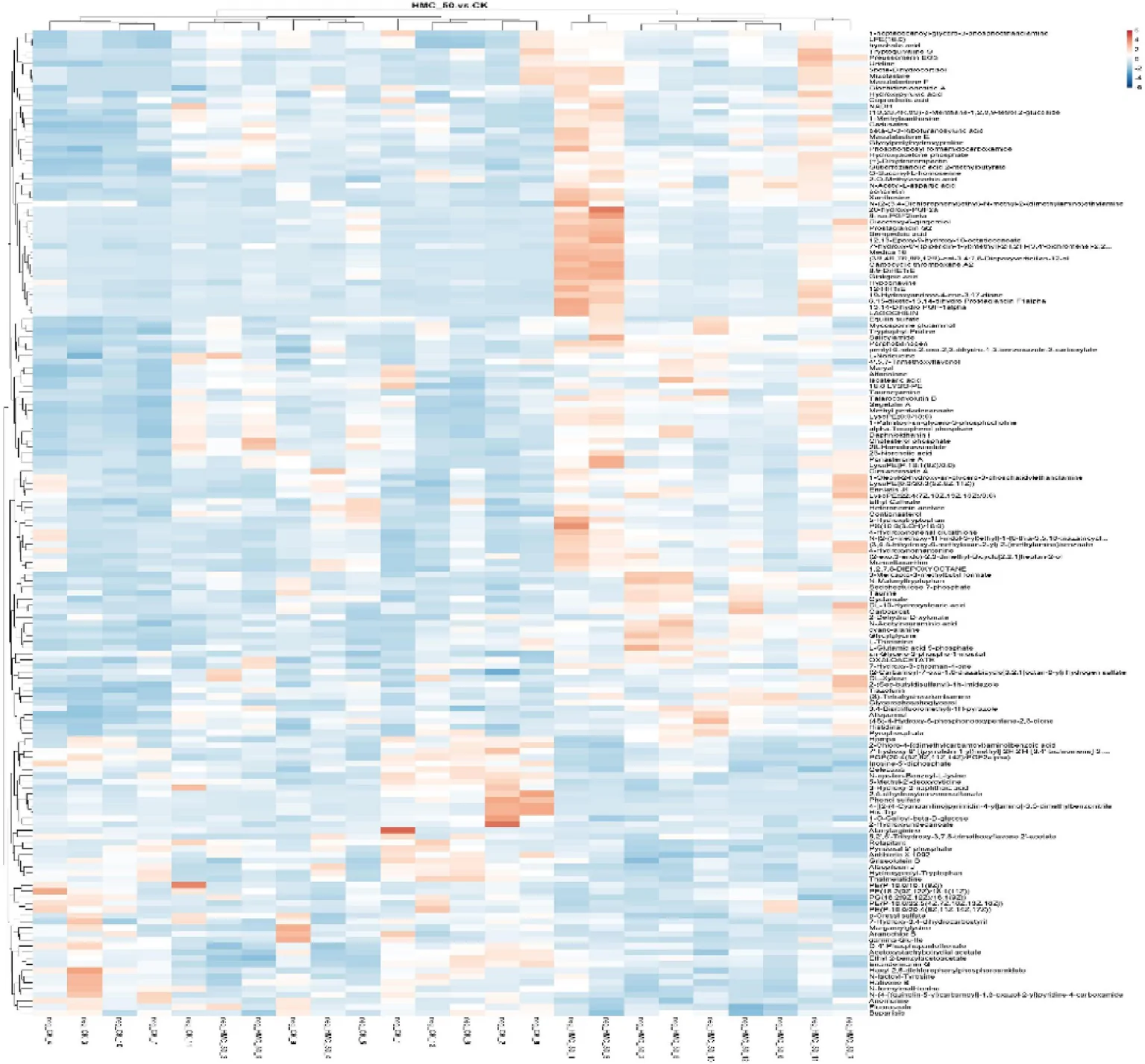
Clustering heatmap analysis of differential metabolites in muscle metabolism of Jinling Yellow chickens fed with HMC: CK represents the control group, HMC_50 represents the HMC 50% group. Blue indicates downregulated expression, while orange/red indicates upregulated expression. The color intensity corresponds to the degree of difference in expression (darker color indicates a more significant difference).

The result was shown in Figure 4, the samples from the HMC50% group and the CK group were clearly separated into two distinct clusters (distinguished by the horizontal blue/orange regions), indicating a significant difference in the overall expression patterns of metabolites between the two groups.

##### 3.2.4.5 KEGG enrichment bubble plot

**Figure 5:**
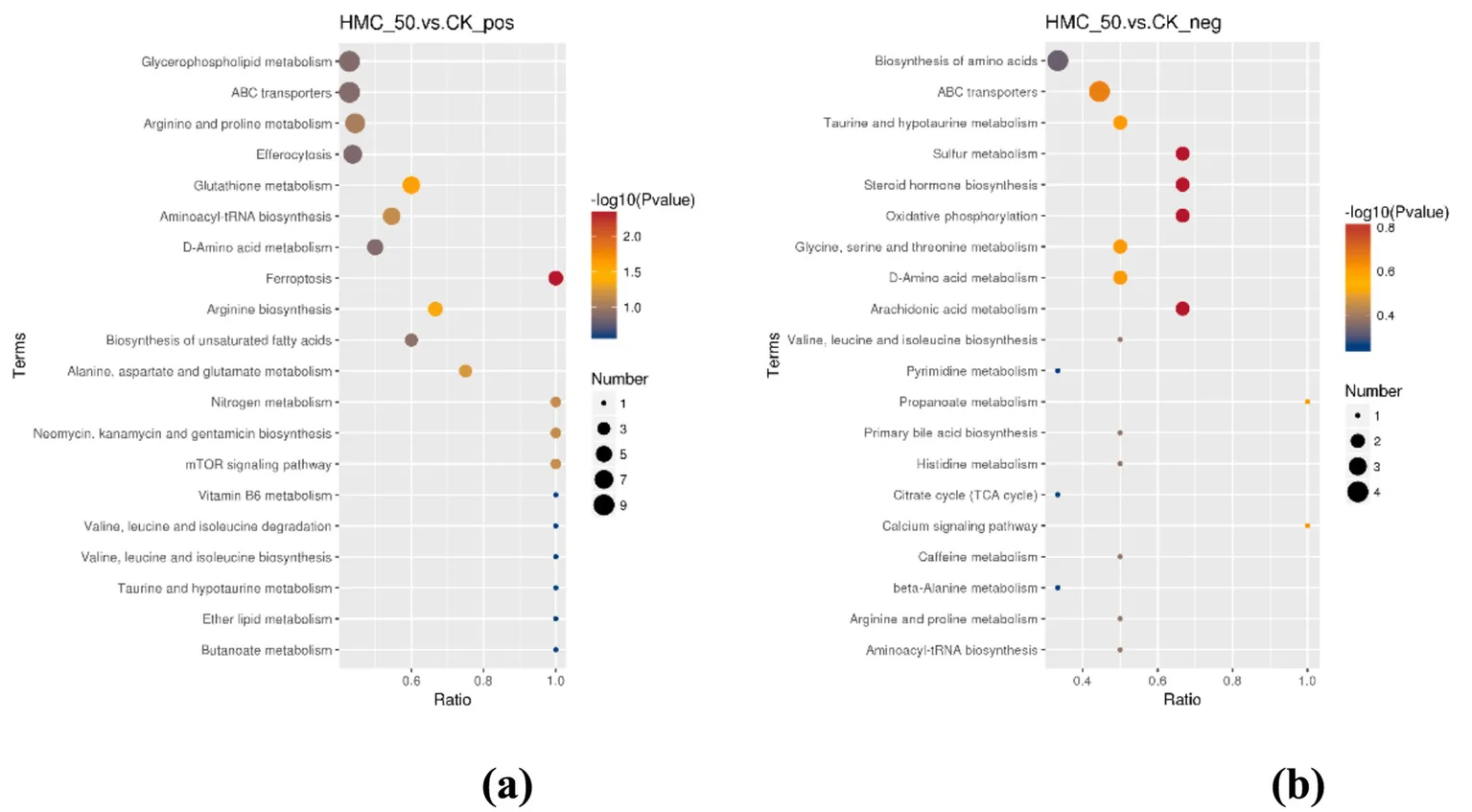
Clustering heatmap analysis of differential metabolites in muscle metabolism of Jinling Yellow chickens fed with HMC: CK represents the control group, HMC_50 represents the HMC 50% group **(a)** KEGG enrichment bubble plot in positive ion mode, **(b)** KEGG enrichment bubble plot in negative ion mode. The x-axis represents the ratio (number of differential metabolites in the corresponding metabolic pathway / total number of identified metabolites in that pathway). A larger value indicates a higher enrichment degree of differential metabolites in that pathway. The color of the dots represents the *p*-value from the hypergeometric test; a smaller value indicates greater reliability and statistical significance. The size of the dots represents the number of differential metabolites in the corresponding pathway; a larger dot indicates more differential metabolites within that pathway.

The results were shown in Figure 5, KEGG enrichment analysis in the positive ion mode (Figure 5a) revealed that the biosynthesis of unsaturated fatty acids and glycerophospholipid metabolism were prominently enriched pathways, with ABC transporters also showing high enrichment, other significantly enriched pathways included arginine and proline metabolism, glutathione metabolism, ether lipid metabolism, and butanoate metabolism; in the negative ion mode (Figure 5b), enrichment analysis revealed another set of significantly altered pathways, the differential metabolites were mainly enriched in specific metabolic and transport pathways, among which those with larger Ratio values included arachidonic acid metabolism, biosynthesis of amino acids, ABC transporters, and taurine and hypotaurine metabolism. In addition, sulfur metabolism, steroid hormone biosynthesis, and oxidative phosphorylation also showed marked enrichment.

### 3.3 Effects of feeding HMC on the cecal microbiota of Jinling Yellow chickens

#### 3.3.1 Alpha diversity analysis

The result of Alpha diversity analysis was shown in Table 9, there were no significant differences in the alpha diversity indices (Chao_1, Dominance, Observed, Pielou_e, Shannon, and Simpson indices) between the two groups (*p* > 0.05).

**Table 9:** Effects of feeding HMC on the alpha diversity of cecal microbiota in Jinling Yellow chickens.

Item	CK	HMC50%	*P*-value
Chao_1	753.21 ± 122.59	715.65 ± 161.52	0.86
Dominance	0.08 ± 0.03	0.07 ± 0.02	0.72
Observed	751.00 ± 121.79	711.83 ± 160.76	0.85
Pielou_e	0.68 ± 0.06	0.65 ± 0.04	0.73
Shannon	6.48 ± 0.70	6.11 ± 0.66	0.71
Simpson	0.90 ± 0.05	0.93 ± 0.01	0.55

#### 3.3.2 Beta diversity analysis

**Figure 6:**
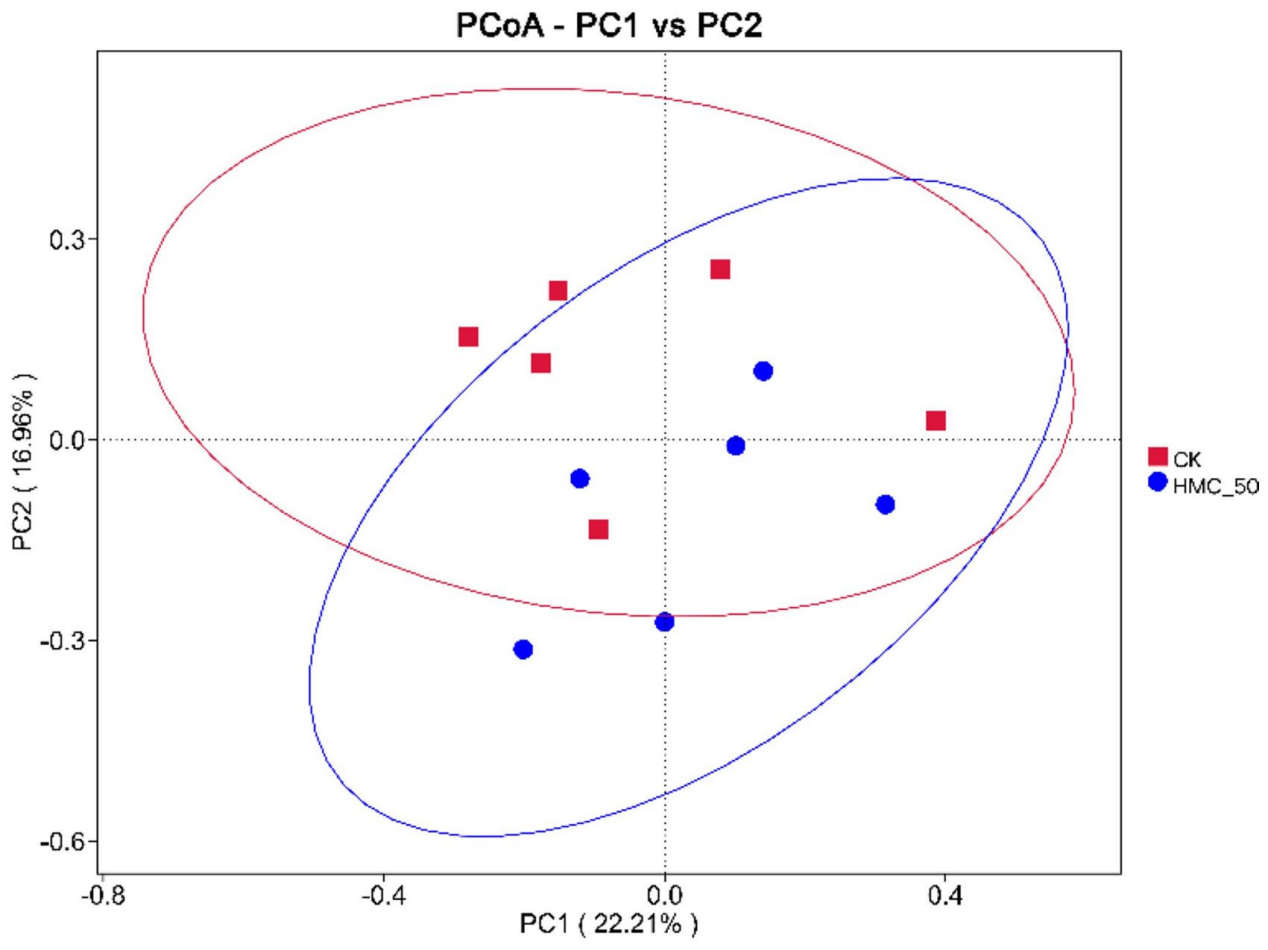
PCoA analysis based on unweighted UniFrac distance: Red (CK) represents the control group, and blue (HMC50%) represents the HMC50% group. The *x*- and *y*-axes correspond to the first and second principal components, with the percentages indicating the proportion of variance explained by each component, each data point denotes an individual sample, and samples within the same group are color-coded identically.

The PCoA analysis based on the Unweighted Unifrac distance (Figure 6) presents different patterns. PC1 and PC2 accounted for 22.21 and 16.96% of the total variance, respectively. On this analytical plane, sample points of the HMC50% and CK groups exhibited an obvious separation trend along the PC1 axis, with the centroid coordinates of the two groups at (−0.45, 0.25) and (0.00, 0.05), respectively.

#### 3.3.3 OTU analysis

**Figure 7:**
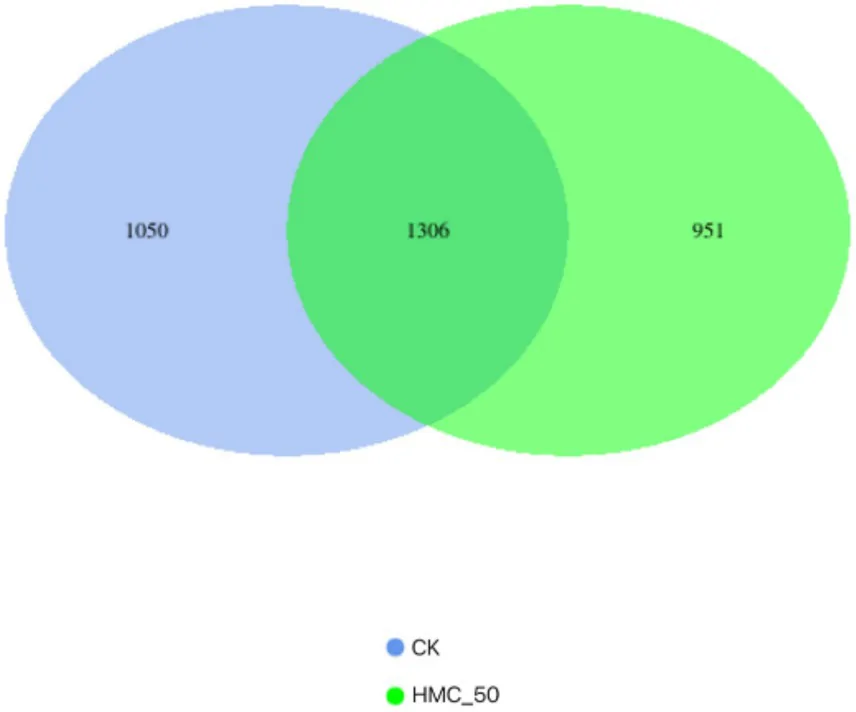
OTU analysis diagram: Each circle represents one group, with blue indicating the CK group and green indicating the HMC50% group. The number in the overlapping region denotes the number of common features shared between the two groups, while the numbers in the non-overlapping sections indicate the features unique to each group.

The result of OTU analysis was shown in Figure 7, the distribution characteristics of cecal microbial OTUs between the HMC50% and CK groups were as follows: a total of 3,307 OTUs were identified, of which 1,306 were shared between the two groups, while 1,050 and 951 OTUs were unique to the CK and HMC50% groups, respectively. These results indicated the presence of a core shared microbiota in both groups, along with group-specific taxa.

#### 3.3.4 Effects of HMC on the relative abundance of cecal microbiota in Jinling Yellow chickens at the phylum and genus levels

**Figure 8:**
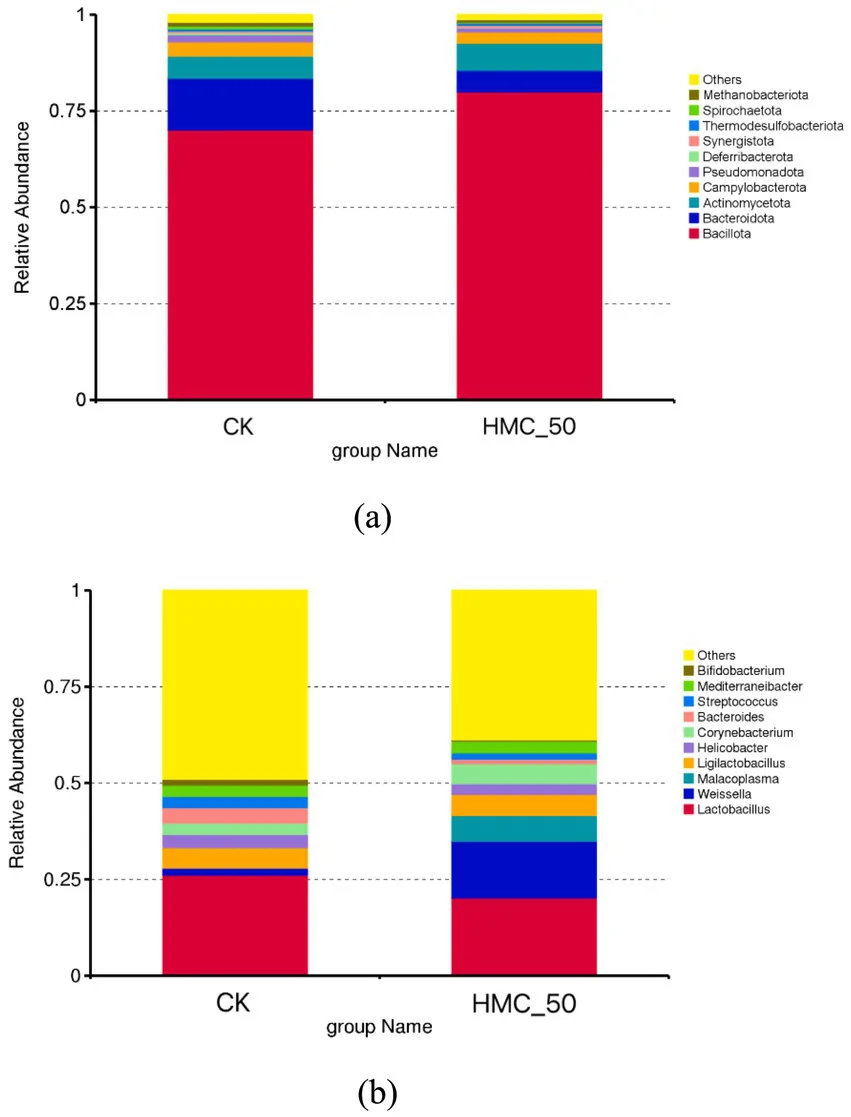
Effects of feeding HMC on the relative abundance of cecal microbiota in Jinling Yellow chickens at the phylum and genus levels: CK represents the control group, HMC_50 represents the HMC50% group. **(a)** Top 10 cecal microbiota with the highest relative abundance at the phylum level; **(b)** Top 10 cecal microbiota with the highest relative abundance at the genus level.

Based on the phylum-level analysis results (Figure 8a), among the top 10 phyla in terms of relative abundance in the cecum of Jinling Yellow chickens, compared with the CK group, the relative abundances of *Bacillota* and *Actinomycetota* in the HMC50% group increased by 14.12 and 23.66%, respectively (*p* < 0.05), whereas the relative abundances of *Bacteroidota*, *Deferribacterota*, and *Spirochaetota* decreased by 58.8, 80.78, and 77.64%, respectively (*p* < 0.05).

Based on the genus-level analysis results (Figure 8b), among the major genera, compared with the CK group, the relative abundances of *Weissella* and *Corynebacterium* in the HMC50% group increased by 726.44 and 78.53%, respectively (*p* < 0.05), whereas the relative abundances of *Lactobacillus*, *Bacteroides*, and *Streptococcus* decreased by 22.65, 69.93, and 45.38%, respectively (*p* < 0.05).

#### 3.3.5 Wilcoxon rank-sum test and LEfSe analysis

**Figure 9:**
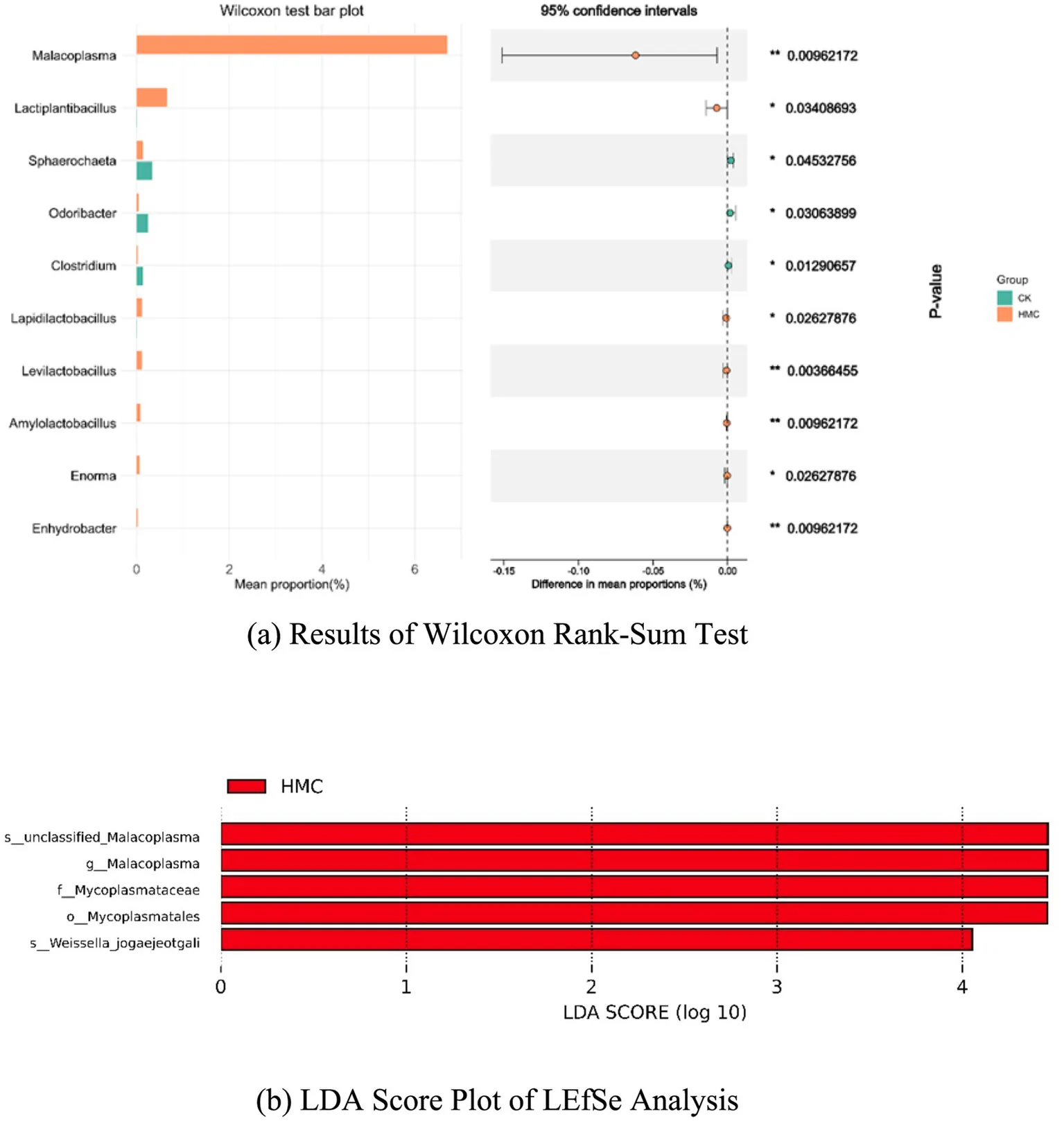
Effects of dietary HMC on the cecal microbiota of Jinling Yellow chickens as assessed by Wilcoxon rank-sum test and LEfSe analysis. **(a)** Results of the Wilcoxon rank-sum test at the genus level: The left panel shows the bar plot of mean relative abundance of genera between the two groups, and the right panel displays the mean differences between groups with 95% confidence intervals. *p*-values are indicated on the right side of the plot (*p < 0.05, **p < 0.01). CK group is shown in green, and HMC50% group in orange; **(b)** LDA score bar plot from LEfSe analysis of the cecal microbiota in Jinling Yellow chickens fed HMC, red bars indicate microbial taxa enriched in the HMC50% group, the horizontal axis represents the LDA discriminant score (log_10_), with a threshold of 2.0, significant biomarker taxa are displayed at the species, genus, family, and order levels.

The results of Wilcoxon rank-sum test are presented in Figure 9a, which revealed that the relative abundances of genera including *Malacoplasma*, *Lactiplantibacillus* and *Levilactobacillus* were significantly higher in the HMC50% group compared with the CK group; the results of LEfSe analysis are illustrated in Figure 9b, showing significant enrichment of the genera *Malacoplasma* and *Weissella* in the HMC50% group.

### 3.4 Correlation analysis between breast muscle fatty acids and cecal microbiota at the genus level in Jinling Yellow chickens

**Figure 10:**
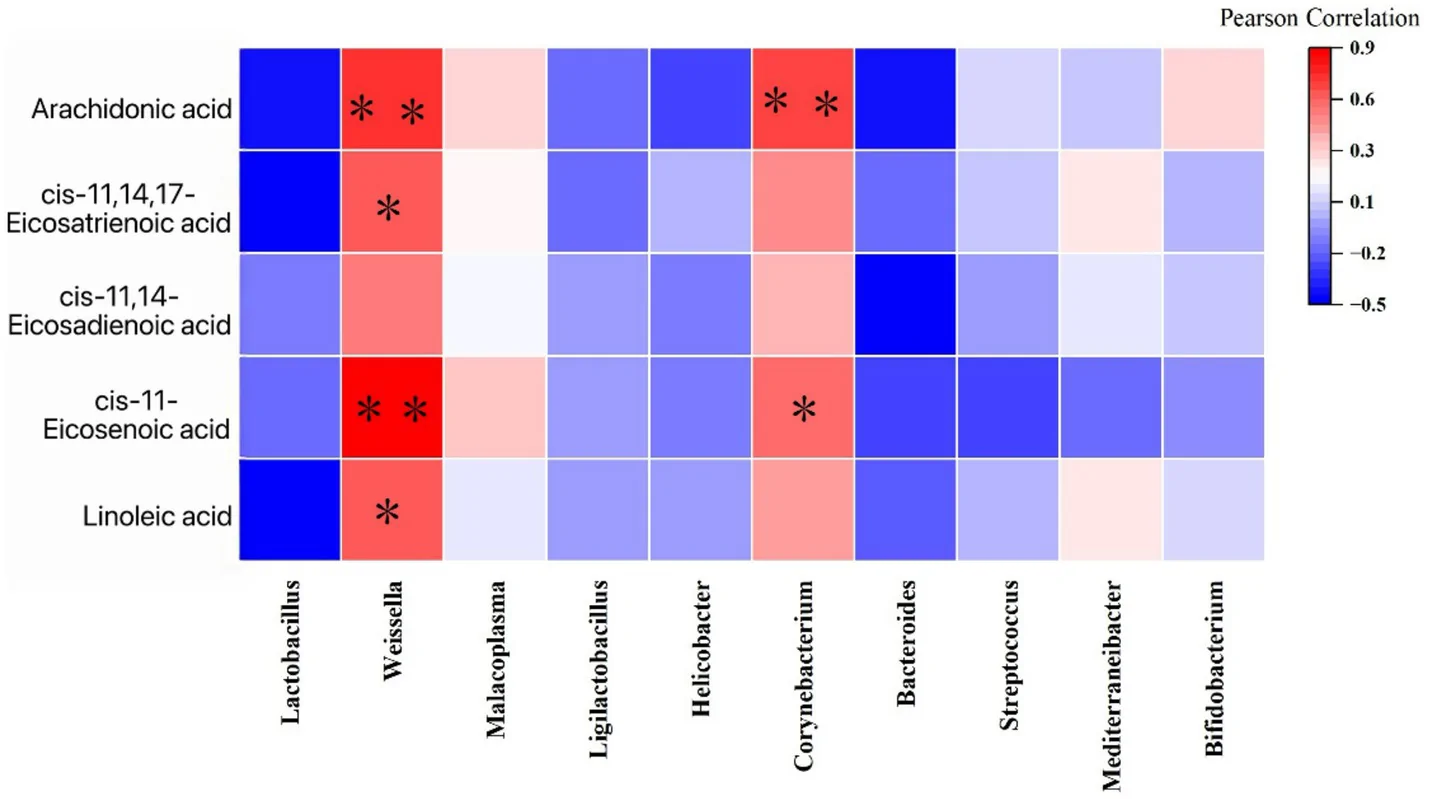
Correlation analysis between breast muscle fatty acids and cecal microbiota at the genus level in Jinling Yellow chickens: This analysis was performed on the five significantly changed fatty acids in the breast muscle and the top 10 microbial genera in terms of relative abundance in the cecal microbiota. “*” indicates significant correlation at the 0.05 level (two-tailed), and “**” indicates significant correlation at the 0.01 level (two-tailed).

The Shapiro–Wilk normality test confirmed that all measured variables in this study followed a normal distribution. Therefore, Pearson correlation analysis was utilized to explore linear correlations between cecal microorganisms and fatty acids in breast muscle, and the results are shown in Figure 10. There were significant correlations between cecal microbiota and breast muscle fatty acids in Jinling Yellow chickens. *Weissella* showed significant positive correlations with cis-11-eicosenoic acid (*r* = 0.647, *p* < 0.05), cis-11-eicosapentaenoic acid (*r* = 0.911, *p* < 0.01), cis-11,14,17-eicosatrienoic acid (*r* = 0.670, *p* < 0.05), and arachidonic acid (*r* = 0.767, *p* < 0.01). *Streptococcus* showed significant positive correlations with cis-11-eicosapentaenoic acid (*r* = 0.596, *p* < 0.05) and arachidonic acid (*r* = 0.689, *p* < 0.05).

## 4 Discussion

### 4.1 Effects of feeding HMC on the performance of Jinling Yellow chickens

This study observed that average daily gain exhibited a declining trend in both the HMC50% and HMC100% groups relative to the CK group. Bhuiyan (16) documented that dietary HMC inclusion suppressed feed intake of broilers prior to 21 days of age, and such reduced feed intake serves as a primary contributor to depressed body weight and ADG. Furthermore, during the dietary adaptation period, intestinal homeostasis exhibited transient fluctuations, which transiently affected nutrient digestion and absorption (17). However, as the intestinal microbiota re-established stability under the new dietary regimen, digestive function was subsequently restored. Consequently, no significant differences in final body weight were observed between the HMC50% or HMC100% groups and the CK group.

In addition, there were no significant differences between the HMC50% and CK groups in eviscerated weight or dressing percentage, indicating that HMC feeding did not adversely affect final production performance or carcass yield. During the initial phase of dietary transition, the high moisture content of HMC may partially dilute the effective nutrient concentration of the feed, nevertheless, as the feeding experimental progressed, cecal microbiota gradually adapted to the modified dietary composition, accompanied by recovered or even compensatory elevated digestive enzyme activity and nutrient absorption efficiency. This enabled the Jinling Yellow chickens to accelerate growth in the later phase, compensate for mid-period losses, and ultimately achieve eviscerated weight and dressing percentage comparable to those of the CK group (18). In summary, dietary HMC supplementation did not adversely affect the final performance or carcass yield of Jinling Yellow chickens, and a 50% inclusion level was identified as the optimal proportion in broiler diets.

### 4.2 Effects of feeding HMC on meat quality of Jinling Yellow chickens

The results showed that, compared with the CK group, the HMC50% group exhibited significantly increased fat content, along with significantly decreased shear force, water-holding capacity, and meat color L* value, indicating that dietary HMC supplementation effectively improved the meat quality of Jinling Yellow chickens. HMC provides higher available energy and promotes intramuscular fat accumulation by upregulating the expression of lipogenesis-related genes and hepatic *de novo* lipogenesis (19). Increased intramuscular fat may disrupt the structural integrity of muscle fibers, thereby reducing shear force and improving tenderness. In addition, a high-energy diet induces post-slaughter muscle glycolysis, leading to a rapid decline in pH, which enhances protein denaturation and alters light reflectance, consequently decreasing water-holding capacity and lightening meat color (20, 21).

The results showed that, compared with the CK group, the HMC50% group had higher total contents of SFA, MUFA, PUFA, and TFA in the breast muscle. Notably, the levels of key functional PUFAs—including linoleic acid, *cis*-11,14-eicosadienoic acid, *cis*-11,14,17-eicosatrienoic acid, and arachidonic acid—as well as the MUFA components *trans*-7-nonadecenoic acid and *cis*-11-eicosenoic acid, were significantly elevated in the HMC50% group compared with the CK group. Dietary fatty acid composition is a key determinant of muscle fatty acid deposition, after fermentation, the starch structure of HMC is disrupted, releasing the bound fatty acids and substantially improving the utilization of unsaturated fatty acids, thereby providing an adequate substrate for muscle fatty acid deposition (22). In this experiment, a relatively pronounced increase was observed in PUFAs, linoleic acid, an essential fatty acid that cannot be endogenously synthesized in broilers, showed a substantial increase in content, which directly reflects its absorption and deposition from HMC. Furthermore, as a precursor for arachidonic acid synthesis, the sufficient supply of linoleic acid also promoted a significant increase in arachidonic acid, further optimizing the nutritional profile of muscle fatty acids (23).

.The present study found that the total essential amino acid content in the muscle of broilers in the HMC50% group was higher than that in the CK group, with significantly increased levels of valine and isoleucine. Fermentation of HMC improves starch structure and amino acid digestibility. Bhuiyan (16) reported that HMC can enhance host nutrient utilization and promote the deposition of amino acids (valine and isoleucine) in muscle by altering starch structure, which is consistent with the results of the present experiment; Vargas (24) confirmed in an experiment with broilers fed corn from different sources that the processing method of corn affects amino acid deposition in broilers, the valine and isoleucine are key substrates for muscle protein synthesis, and their increased content can improve the nutritional value of meat, thereby enhancing meat quality. Furthermore, there were no significant differences in non-essential amino acids between the HMC50% group and the CK group, indicating that the addition of 50% HMC did not disrupt the overall amino acid balance in the muscle of this broiler breed. In summary, feeding 50% HMC can significantly improve the meat quality of Jinling Yellow chickens.

### 4.3 Effects of feeding HMC on muscle metabolism in the breast muscle of Jinling Yellow chickens

In this study, both PCA and PLS-DA results showed a clear separation between the HMC50% group and the CK group in multidimensional space, demonstrating that the HMC-supplemented diet substantially reshaped the global metabolomic signature of breast muscle in Jinling Yellow chickens. Zhou (25) reported that alterations in dietary nutrient composition can modulate the cecal microbial community and intestinal nutrient absorption, thereby influencing the fatty acid profile and metabolite abundance in muscle through their involvement in fatty acid synthesis and metabolism, this is consistent with our findings, reflecting the regulatory effect of diet on muscle metabolism.

In this study, a total of 405 significant differential metabolites were identified, including 243 in POS and 162 in NEG, which may be attributed to the role of HMC in promoting nutrient absorption and metabolite synthesis. Buweiaizhaer (26) reported that, owing to characteristics such as structural softening and dissociation of the binding bonds between starch and protein, HMC can significantly improve the digestibility and utilization of dietary crude protein and crude fat. Metabolomics analysis further revealed upregulation of LysoPC, free amino acids, and their derivative metabolites in the body. Furthermore, Xu (27) reported that taurine enhances the antioxidant capacity of broiler breast muscle through activation of the Nrf2 signaling pathway, thus contributing to improved meat quality. In addition, the distinct separation of the two groups in the cluster heatmap provided further support for the coordinated expression profiles of the differential metabolites.

KEGG pathway enrichment analysis in the present study revealed that differential metabolites were predominantly enriched in pathways related to amino acid biosynthesis, unsaturated fatty acid biosynthesis and glycerophospholipid metabolism, which were closely associated with the improvement of meat quality in HMC-fed Jinling Yellow broilers. Enrichment of the amino acid biosynthesis pathway accounted for the accumulation of essential amino acids, such as valine and isoleucine, in muscle tissue, thereby enhancing the nutritional quality of the meat. Additionally, the ABC transporter pathway was highly enriched in both positive and negative ion modes, suggesting that HMC may modulate transmembrane transporter activity, influence metabolite exchange between muscle cells and their microenvironment, and ultimately facilitate nutrient transport to muscle tissue (22). Arginine is a functional essential amino acid for broilers, serving multiple physiological roles. Studies have demonstrated that an appropriate dietary level of arginine can increase leg muscle yield and improve meat tenderness and flavor quality (28); Glutamate as a vital flavor-active substance in chicken meat and exerts a crucial influence on meat taste quality. He et al. reported that glutamate serves as the primary metabolic fuel for intestinal epithelial cells of young chicks, with a far higher oxidation rate than glucose and fatty acids (29); glutathione is a crucial non-enzymatic antioxidant within cells, playing a key role in scavenging reactive oxygen species and maintaining cellular redox homeostasis. Studies have demonstrated that dietary supplementation with guanidinoacetic acid can alleviate muscle oxidative damage induced by pre-slaughter transport stress in broilers through the modulation of glutathione metabolism (30). Collectively, dietary HMC supplementation may enhance the nutritional value, flavor characteristics and antioxidant capacity of broiler breast muscle by promoting the accumulation of amino acids including valine, arginine and glutamate, thereby improving overall breast meat quality.

Although the biosynthesis of unsaturated fatty acids and glycerophospholipid metabolism did not reach statistical significance, their relatively high Ratio indicated that the metabolic mechanisms within these pathways were substantially affected, the enrichment of the biosynthesis of unsaturated fatty acids pathway was associated with a significant increase in muscle PUFAs content, and PUFAs are critical determinants of meat tenderness and nutritional value; the lysophosphatidylcholine (LysoPC) can strengthen the stability of muscle cell membranes and improve water-holding capacity of meat, thus reducing post-slaughter water loss (31).

In summary, HMC enhances nutrient utilization and promotes the accumulation of nutrients in broiler muscle metabolism by enriching amino acids and PUFAs to modulate metabolic enzyme activity, thereby improving meat quality.

### 4.4 Effects of feeding HMC on the cecal microbiota of Jinling Yellow chickens

In this experiment, no significant differences were observed in alpha diversity indices (e.g., Chao1 and Shannon) between the CK group and the HMC50% group; PCoA analysis based on unweighted UniFrac distance revealed a clear separation of the two groups along the PC1 axis; OTU analysis showed that the two groups shared 1,306 OTUs, while the CK group and the HMC50% group had 1,050 and 951 unique OTUs, respectively. These results indicate that feeding HMC did not alter the basic characteristics of microbial species richness or evenness, but significantly reshaped the community structure of the cecal microbiota. Yoon (32) confirmed that corn-based diets can significantly alter the beta diversity of cecal microbiota in broilers, and that the clustering differences in microbiota caused by different grain sources are mainly attributed to the physicochemical properties of the grains themselves, such as starch and energy content. Liu (33) demonstrated that the intestinal microbiota of broilers can regulate host lipid metabolism through their metabolites, short-chain fatty acids (SCFAs). Among the SCFAs, propionate and butyrate are closely associated with fat deposition. Therefore, the substantial changes in the cecal microbiota provide a structural basis for the increased fatty acid content in muscle, suggesting that functional alterations of the microbiota may participate in the regulation of host lipid metabolism via the SCFAs pathway.

At the phylum taxonomic level, *Firmicutes* exhibited the most prominent compositional shift, whereas a marked reduction in Bacteroidota drove a substantial rise in the *Firmicutes*-to-*Bacteroidota* (*F*/*B*) ratio. Xiang (34) validated this relationship via 16S rRNA gene sequencing and metagenomic profiling across 400 broilers, establishing that depleted *Bacteroidota* alongside enriched *Firmicutes* (reflected by an elevated *F*/*B* ratio) are significantly and positively correlated with abdominal fat deposition in broilers. This finding is consistent with our results, suggesting that the elevated *F*/*B* ratio offers a mechanistic explanation at the phylum level for the significant increases in monounsaturated and polyunsaturated fatty acid contents in broiler muscle. At the genus level, the relative abundance of *Weissella* was markedly increased in the HMC50% group. As a potential probiotic, metabolites of *Weissella* may participate in host lipid regulation (35). Therefore, its increased abundance may promote the deposition of PUFAs such as linoleic acid and arachidonic acid in muscle through mechanisms including the secretion of bile salt hydrolase and the conversion of PUFAs. In addition, the relative abundances of *Lactobacillus*, *Bacteroides*, and *Streptococcus* were significantly reduced in the HMC50% group, and the decrease in *Lactobacillus* may alleviate its inhibitory effect on fat deposition. Wang (36) demonstrated that *Lactobacillus* can reduce fat deposition by regulating the expression of lipid metabolism-related genes. Meanwhile, as a genus associated with lipid reduction, the decreased abundance of *Bacteroides* also promoted fatty acid deposition (37, 38). In addition, dietary fat composition can significantly affect the colonization level of *Streptococcus*, and changes in its abundance may indirectly participate in lipid regulation by influencing bile acid metabolism (39).

Results of Wilcoxon rank-sum test and LEfSe analysis demonstrated that the relative abundances of genera including *Malacoplasma*, *Lactiplantibacillus*, *Levilactobacillus* and *Weissella* were markedly elevated in the cecum of the HMC50% group relative to the CK group. Such alterations in microbiota composition may be closely associated with the physicochemical properties of HMC. The relatively HMC of HMC renders it susceptible to fermentation during storage, leading to the production of organic acids such as lactic acid and acetic acid (40). Yin (41) demonstrated that prolonged corn storage alters starch pasting properties and soluble sugar concentrations, further modulating the availability of fermentable substrates for intestinal microbes these physicochemical characteristics may provide a more favorable growth environment for lactic acid bacteria. *Weissella* is one of the prevalent lactic acid bacterial genera in the intestinal tract of poultry. Studies have shown that the genome of *Weissella jogaejeotgali* harbors genes associated with fatty acid synthesis and degradation as well as glyceride metabolism (42). Accordingly, the enrichment of *Weissella* in the HMC50% group may be attributed to its potential function in regulating host fatty acid metabolism.

In summary, feeding HMC promoted the deposition of fatty acids in the muscle by reshaping the cecal microbiota community structure of broilers, thereby increasing the fatty acid content in broiler muscle.

### 4.5 Correlation analysis between breast muscle fatty acids and cecal microbiota at the genus level in Jinling Yellow chickens

Pearson Correlation analysis revealed that the cecal genus *Weissella* was significantly positively correlated with multiple PUFAs, including cis-11-eicosenoic acid, cis-11-eicosapentaenoic acid and arachidonic acid. Meanwhile, the genus *Streptococcus* also exhibited significant positive correlations with several PUFAs (cis-11-eicosapentaenoic acid and arachidonic acid). These findings suggest that shifts in the abundance of broiler cecal microbiota induced by HMC are closely linked to the accumulation of PUFAs in breast muscle. As a beneficial genus belonging to Lactobacillaceae, *Weissella* may facilitate the hydrolysis and conversion of dietary PUFAs via secreting metabolic enzymes such as lipases and esterases. Lactobacilli can produce bile salt hydrolase to degrade bile acids; this enzyme participates in regulating lipid anabolism and accelerating lipid catabolism, thereby modulating host lipid metabolism (43); the genus *Streptococcus* is closely associated with bile acid metabolism and lipid regulation under high-fat dietary conditions. Luo (44) verified that supplementation with *Streptococcus* correlates with altered bile acid profiles and improved lipid metabolic indicators, including reduced serum triglyceride and cholesterol concentrations, implying that this genus participates in lipid regulation. Collectively, *Weissella* and *Streptococcus* may influence intestinal fatty acid absorption and hepatic lipid metabolism in Jinling Yellow chickens through multiple pathways, including bile salt hydrolase production, PUFAs bioconversion, and bile acid metabolism, thereby modulating the deposition of PUFAs in muscle. This pattern may be correlated with the changes in muscle PUFA content observed in the present study.

## 5 Conclusion

The results of this study indicated that replacing normal corn with 50% HMC significantly improved meat quality (reduced shear force and increased water-holding capacity) and promoted the accumulation of polyunsaturated fatty acids and essential amino acids (e.g., valine and isoleucine) in the breast muscle, without compromising the growth performance of Jinling Yellow chickens; HMC may synergistically regulate nutrient deposition in the breast muscle by activating multiple metabolic pathways, including the biosynthesis of unsaturated fatty acids, glycerophospholipid metabolism, and amino acid biosynthesis, while also remodeling the cecal microbial community structure (enriching beneficial bacteria such as *Weissella*); Pearson correlation analysis revealed close correlations between cecal microbial genera and fatty acids in breast muscle of Jinling Yellow broilers. In summary, dietary inclusion of 50% HMC improved growth performance and meat quality in broilers, thereby providing a theoretical basis and practical reference for the resource-efficient and low-cost application of HMC in broiler production.

## Data Availability

The data presented in the study are deposited in Figshare, DOI: 10.6084/m9.figshare.33176129.
